# Role of Clinical Neuropsychologists in Awake-Craniotomy

**DOI:** 10.4103/0028-3886.319237

**Published:** 2021-05-01

**Authors:** Vasudha H Hande, Harini Gunasekaran, Shantala Hegde, Abhinith Shashidhar, Arivazhagan Arimappamagan

**Affiliations:** 1 Clinical Neuropsychology and Cognitive Neuro Science Center, Department of Clinical Psychology, National Institute of Mental Health and Neurosciences, Department of Neurosurgery, National Institute of Mental Health and Neurosciences, Bengaluru, Karnataka, India

**Keywords:** Awake craniotomy, clinical neuropsychologist, intraoperative evaluation, neuropsychological evaluation, psychological intervention

## Abstract

**Background:**

Awake craniotomy encompasses surgical resection of focal zone of neurological impairment, using intraoperative functional zone mapping. The strength of the procedure is maximum retention of functionally significant zones to ensure better function and quality of life outcomes in patients. A clinical neuropsychologist plays a vital role in profiling the patient’s cognitive and psychosocial functioning as well as increasing the efficacy of functional zone mapping procedures.

**Objective:**

Aim of this article is to summarize the literature on the role of clinical neuropsychologists in awake craniotomy and underscoring the need for establishing standardized operating procedures for neuropsychologists in awake craniotomy highlighting experiential anecdotes from a tertiary care facility.

**Materials and Methods:**

A review of articles that elucidate the role of clinical neuropsychologists was done and summarized to highlight the role of clinical neuropsychologists. An attempt was made to explain the implementation of this role in regular clinical practice at a tertiary care facility.

**Results:**

The role of a clinical neuropsychologist is highly crucial at pre-/during and postawake craniotomy, and has a significant bearing on the overall psychological outcome of the individual. The need for a standardized protocol to unify practice and increase the efficacy of the awake-craniotomy procedure is put forth. Recommendations for future directions in research to increase the scope of neuropsychologists in awake craniotomy have also been made.

Awake craniotomy is performed with intraoperative electrical stimulation brain mapping with conscious patients. It is now considered as a gold standard when the patient has a focal zone of neurological impairment (such as a tumor or focal epileptogenic region) in functionally sensitive regions of the brain. The primary goal of awake craniotomy is to evacuate the impaired cells/tumor as much as possible without damaging the crucial cognitive functions and functions that may be crucial for the overall functioning of the patient. With balancing maximum resection of the impaired cell structures while preserving functional zones, this form of surgical intervention paves way for a better prognosis. Pre-/intraoperative and postsurgical assessment of neurocognitive functions is essential in determining the outcome and prognosis.^[1–6]^

The typical process of awake craniotomy involves an asleep–awake–asleep protocol. In the initial asleep phase, the patient is put under a general anesthetic followed by local anesthesia in the scalp region while his/her vitals are continuously monitored. Next, a surgical incision of the scalp is made, and the skull is removed. Following this is the incision of the meninges and stitching it to the side, to finally give the neurosurgeon access to the cortical regions of the brain. The awake phase of the surgery begins when neuroanesthesiologists, while carefully monitoring the patient and continuing to keep him/her under the local anesthesia to manage pain, bring the patient back to consciousness. The aim of this phase is to use intraoperative direct electrical stimulation (DES) to identify and carefully map the functionally positive regions in and around the zone of excision in order for it to be preserved. A region is considered functionally positive when the DES of the site elicits an abnormal response (motoric, e.g., twitching, and sudden sensory loss/language, e.g., anomia and paraphasia). Such regions are marked and surgical excision is followed with careful monitoring. The final asleep phase begins when the patient is carefully put back under sedation to facilitate the neurosurgeon to close the surgical site, i.e., suturing the meninges and the scalp.^[5]^

Undergoing surgery in general, neurosurgery in specific, can be a traumatic experience for the patient.^[7]^ Awake craniotomy adds to the trauma of the patient—they are responsible for guiding the surgical procedure during intraoperative mapping through their speech and motor responses. A well-informed and emotionally stable patient is likely to show better cooperation and coping during the surgery, and have a better prognosis.^[7]^

In view of this, a clinical neuropsychologist has a major role prior, during and post awake craniotomy for assessment as well as intervention in cognitive and psychosocial domains.^[8]^ The aim of this paper is to discuss the role of clinical neuropsychologists in awake craniotomy, highlighting experiential anecdotes of the procedure at a tertiary care facility—National Institute of Mental Health and Neurosciences (NIMHANS), Bengaluru, India. Along with reviewing other published articles, the authors provide recommendations for establishing standard operating protocols for incorporating these practices uniformly.

## Role of Neuropsychologist Before Surgery

### Assessment

The evaluation begins with collecting a thorough history from the patient. This includes establishing the history of the neurosurgical condition, relevant personal history of illness, screening for psychiatric conditions, evaluating patient’s premorbid cognitive ability, and personality. Psychiatric conditions like drug abuse, schizophrenia, anxiety disorder, and intellectual disabilities are contraindications for awake surgeries. Besides the clinical interview, the use of standardized instruments for screening could help establish uniformity in practice.

Some brief clinicians rated rating scales that can be used are as follows.

- Modified Mini Screen.^[9]^
- Mini Screen.^[10]^

Anxiety and distress are common secondary symptoms of severe neurological conditions. Evaluation of these symptoms requires tools sensitive to emotional and cognitive manifestations of these conditions since the somatic manifestation can be masked by the primary neurological condition. Examples of such sensitive tool, with the advantage of being brief and clinician rated are as follows.

- Hospital Anxiety and Depression Scale.^[11]^
- Patient Health Questionnaire.^[12]^

It is important to evaluate the patient’s personality factors, such as temperament, typical coping styles to determine factors, precipitating as well as protective to the patient’s ability to cope through the surgery. The neuropsychologist should also evaluate patients’ access to resources for support—emotional, familial, financial, and occupational.

A thorough evaluation of the patient’s cognitive functioning to obtain the functioning profile of the patient using tests from a standardized Neuropsychological Battery and standardized tests. If history is suggestive of intellectual difficulties, it is better to first do an objective evaluation of IQ to rule out mental retardation. It is also important to screen for handedness of the patient with a gold standard tool such as Edinburgh Handedness Inventory,^[13]^ which helps understand cerebral dominance for functioning in the individual. This is important in interpreting the overall functional profile of the individual.

After establishing the patient’s baseline neuropsychological profile, a few selected tests covering the domains of functioning corresponding to the relevant site of impairment and suitable for administering intraoperatively are chosen and a baseline of these tests are again obtained a day before the surgery. The choice of tests is maintained to cover the domains of orientation, comprehension, attention, fluency, working memory, language, and visual and memory with the modalities of response being both motoric and spoken.

### Experience at NIMHANS

At NIMHANS, a patient posted for awake surgery undergoes a detailed neuropsychological assessment using standardized neuropsychological tests adapted and suitable for Indian population. Norms for Indian population based on age, education, and sex are available. NIMHANS Neuropsychology Battery^[14]^ is typically used. Selected tests from the battery of tests developed by Dr. Mukundan *et al.,*^[15]^ Western Aphasia Battery (WAB)—the ICMR adaptation suitable for Indian population,^[16]^ Wechsler Memory Scale III^IND^ (WMS III^IND^),^[17]^ and Addenbrooke’s Cognitive Exam (ACE)^[18]^ are often used in addition. We have also used specific tests tapping functions such as music and rhythm perception in cases where the loss of these functions can be debilitating to a patient’s functioning postsurgery.^[4]^ Trained clinical psychologists carry out these assessments a few days before the surgery. Refer to Table 1 for some of the commonly used tests at NIMHANS presented domain wise.

### Interventions

It is important to understand the expectations, concerns, and fears of the patients to prepare them for surgery. Often patients are surprised when they first hear about being awake during brain surgery.^[19]^ Patients are concerned about self-preservation, surgical outcomes, and possibility of complications such as death and permanent disability.^[20]^ Preoperative counseling is indispensable for preparing them better for the surgery. Patients and their families need to be psychoeducated about the procedure, the surgical team has to be introduced to the patient, and rapport has to be built with the entire operative team before the surgery.

### Experience at NIMHANS

The aim of testing during awake surgery is to ensure the preservation of the existing level of functionality in the patient. The baseline neuropsychological assessment with the patient is normatively compared to sensitively yield a profile of the patient’s functions that are impaired as well as those preserved. Out of this profile, the tests administered at baseline along with test items that yield to administration intraoperatively, for functions that are preserved (as seen by above -1SD cutoff performance on Neuropsychological Battery) and associated particularly with the areas at and around the site of tumor are chosen. Evaluation of performance on these tests is clinically evaluated and compared with the patient’s performance at different points—starting a day before surgery.

A day before the surgery, the patient is exposed to tests to be administered intraoperatively and educated regarding the same. This is extremely important as it reduces the anxiety of the patient and gives the examiner an accurate understanding of the patient’s abilities and estimate of performance during the awake surgery. In case of extremely poor performance on any test, it is excluded from the intraoperative protocol.

The clinical psychologist evaluates and addresses emotions and concerns of the patient. Relaxation techniques like deep breathing may be taught to the patient, when necessary. Patients are given information related to the surgical procedure, techniques, patient positioning and discomfort, possible pain associated, and the duration of procedure besides possible long-term side effects of the procedure, if any. It is necessary to explain to the patient in prior the importance of his/her cooperation during the awake surgery. It is observed that assurance of the therapist’s presence during the surgery ensures better coping intraoperatively.

The process of psychoeducation begins with the assessment of the patient’s individual psychological and psychosocial factors. Level of anxiety, expectations, and fears regarding the risks and pain of the procedure, postsurgical outcome expectations, concerns regarding financial, occupational, and inter- and intrapersonal challenges postsurgery are all assessed. A systematic review by Hom, Kaneshiro and Tsui *et al.,* on presurgical psychoeducation, suggests that strategies that help evaluate and positive patient’s estimates of their recovery and ability to tolerate pain yielded better postsurgical recovery rates. Similarly, they identified that an open communication, where the patients are given opportunity to be involved in the process and given the space to express their doubts and concerns about procedure, yields to better postsurgical satisfaction and recovery.^[25]^

In lieu of this, the typical process of psychoeducation is tailored to the needs of the patient. All patients undergoing the surgical process are explained the procedure and the risk involved in a language that they understand with terms that are appropriately simplified depending on their background of education. It is recommended that they are given support to the extent as needed. This may be brief psychoeducation to supportive/cognitive-behavioral sessions, depending the primary concerns for the patient. Debriefing and support are extended postoperatively as well.

Patients could also be benefited from the development of simple IEC booklets regarding the typical procedures, level of pain, risks involved, and support services available for them through the process.

## Role of Neuropsychologist During the Surgery

### Assessment

A predetermined sequence of conducting the assessments can help quicken the process of determining the functionally positive zones during the intraoperative DES.

### Experience at NIMHANS

During the surgery, a clinical psychologist assists the team in the mapping of cognitive functions. We also prepare materials that can be used for testing various cognitive functions. Often these test materials are case-specific. Expressive speech is tested by enumeration tasks like reciting numbers, reciting days of week, names of months, etc. The sensory aspects of speech and other neurocognitive functions are tested by tasks like picture naming, picture description, reading a sentence, famous face identification, etc. Other tests include verbal fluency, simple calculations, working memory task, visuospatial cognition visual field, facial recognition, and emotion recognition test. Depending on the area of interest, other sensory functions such as olfaction are also assessed using aromatic substances like coffee beans/vanilla beans/peppermint. Refer to Table 2 for some of the commonly used tests during inter-operative phase in our Institute.

A baseline is repeated right before the beginning of the asleep- phase and at the beginning of the awake phase. Following this, the choice and sequence of the tests can be varied depending on the area under DES (e.g., language tasks when the eloquent areas are under DES versus executive tasks when anterior frontal regions are under DES). The testing is synced with the standard protocol for DES.

Post the mapping and the beginning of excision, the testing across domains is repeated at regular intervals, with adequate breaks to maintain the balance of keeping the patient comfortable, motivated, and alert for the whole process. A careful observation for signs of anomia, paraphasia, and/or speech arrest is maintained.[21] A final repeat of the whole baseline is done just before the closing. Care is taken to choose items variably as well as change the order of presentation to counter for practice effects.

### Intervention

When the patient has been positioned for awake surgery, a therapist should be by his/her side. The awake phase puts multiple demands on the patient—there is discomfort for the patient, along with fear/anxiety of being in an operation theatre and they are expected to stay alert and respond to cognitive tasks amidst this. It helps for the patient to engage constantly with just one primary contact and the therapist often becomes this point of contact. Thus, the neuropsychologist juggles to continue assessment while continuously checking and ensuring patient’s comfort as much as possible working with the rest of the team.

### Experience at NIMHANS

Assuring patient’s comfort, keeping him/her awake during the period of DES and neuropsychological evaluation during surgery and recording patient’s response on a minute-by-minute basis are highly crucial. Presurgery psychoeducation, preparing the patient for the possible discomfort during the awake phase, and during the awake phase, statements validating the patient’s discomfort and appreciating/encouraging their strength, as well as reassuring them with emotional/problem-solving focused coping statements depending on the patients individual coping style has been found helpful to support the patient through the process.

Intraoperative management of anxiety in a patient poses obvious challenges in the form of restricted access to the patient with the ongoing procedure and the demands of the same on patients. Intraoperatively besides use of relaxation techniques based on mindfulness and/or breath control, the use of music-based relaxation can also be explored. Gestures of reassuring dialogue with patients especially after having formed a good therapeutic alliance and/or holding their hands goes a long way in reducing the patient’s level of anxiety intraoperatively.

## Role of Neuropsychologist After the Surgery

### Assessment

Postsurgical assessments begin with a repeat of the baseline assessments once the patient is shifted after recovery. Debriefing of the cognitive status can be given emphasizing on cognitive effects of craniotomy procedure and recovery of the same over time. Repeat neuropsychological evaluation may be attempted at follow-up, to determine a pre–post comparison profile to document the extent of cognitive changes. Besides determining the cognitive outcome, these assessments help determine the goals of cognitive rehabilitation. Serial follow-up at regular interventions can help track changes.^[6]^

### Experience at NIMHANS

Typically, a repeat of the measures used intraoperatively is done before discharge. The first repeat neuropsychological evaluation is done typically around the six months follow-up postsurgery, to counter practice and recovery-related effects on the neuropsychological profile. Attempts are made to repeat evaluations at every 6-month to 1-year interval.

### Interventions

A day after the surgery feedback about patient experiences can be taken and their queries about the recovery process addressed. Pain management techniques can be taught to the patient. Regular follow-up assessments of cognitive functions need to be carried out at 1 week, 1 month, 4 months, and 12 months[22] to monitor progress. Holistic neuropsychological rehabilitation has to be planned.

### Experience at NIMHANS

At the time of repeat assessment before discharge, a debriefing session is held with the patient. The patient is asked for feedback of their experience and their concerns about the recovery process addressed. Psychoeducation and supportive techniques are the choices of intervention here again. Postoperatively, there are possibilities of exploring several therapeutic strategies including supportive psychotherapy, cognitive-based interventions, pain management intervention, and mindfulness-based interventions—depending on the need of the patient. In addition, home-based cognitive retraining strategies for attention, memory, and executive functions are taught to the patient to address the neurocognitive deficits that are observed. These are modified and monitored at the time of follow-up. Based on the need of the patient, a holistic approach of neurorehabilitation is provided.

### Standardizing the Role of a Clinical Psychologist

The role of the clinical neuropsychologist during awake-craniotomy is definitely crucial. However, the standardized procedure is yet to be put in place, especially in our country. Standardizing the procedure is necessary for efficacy, the scope for process-related research to curate the procedure. Reflecting on the challenges faced by the authors in setting up standard methods of practice within the institution, following are some recommendations of aspects that need to be covered in setting up standardized operating procedures for clinical neuropsychologist in awake craniotomies.

- Development and standardization of assessment tool kit to be administered during the surgery—this toolkit should contain simple, short tests to assess speech and various cognitive functions depending on the region involved in surgery, as tabulated above. Care needs to be taken to choose instruments that are applicable across varied literacy levels and available across different languages. The development of parallel forms for all the instruments used is vital to increase the frequency and accuracy of follow-up evaluations.
- A standardized interview schedule could be developed to address patient’s concerns and understand his/her knowledge about surgery and prognosis.
- Development of presurgery intervention, including relaxation techniques (a week-long session of relaxation procedures like meditation, muscle relaxation, and/or breathing exercises can be considered).
- Research focus on pre-post neuropsychological assessments to understand the long-term cognitive side effects of awake craniotomies is necessary so that interventions can be tailored accordingly.

## Figures and Tables

**Table 1:** Shows the domainwise neuropsychological assessments commonly used at NIMHANS

Domain	Subdomain	Neuropsychological Assessment
Orientation		MMSE^[10]^
Comprehension		Auditory/visual commands-verbal/performance-based responses. Simple- and complex-token test^[14]^/ Mukundan’s battery^[15]^/WAB^[16]^/MMSE^[10]^/ACE^[18]^
Speed	Motor speed	Finger tapping test^[14]^
	Processing speed	Digit Symbol Substitution^[14]^
Attention	Focused	Color Trails-1^[14]^
	Sustained	Digit Vigilance Test^[14]^
	Shift	Color Trails-2^[14]^
Working memory	Verbal	Digit span test^[14]^
		Verbal N Back^[14]^
	Visual	Spatial Span^[14]^
Fluency	Category	Animal Names Test^[14]^
	Phonemic	Controlled Oral Word Association Test^[14]^
Planning		Tower of London^[14]^
Set shifting		Wisconsin Card Sorting Test^[14]^
Response inhibition		Stroop test^[14]^
Language	Speech and Language	Western Aphasia Battery^[16]^
	Overall	Controlled Oral Word Association Test
	Fluency	Animal Names Test
	Reading	Binet Kamat Test Passage^[23]^
	Writing	Copying, Dictation and Writing a paragraph on a general topic.
Memory	Verbal	Rey’s Auditory Verbal learning test^[14]^; Verbal paired associates test^[17]^; Logical Memory Test^[17]^
	Visual	Complex Figure Test^[14]^
Visuospatial		Bender Gestalt test^[24]^; Cube drawing; Complex Figure Test^[14]^
Parieto-occipital focal signs		Tests of naming and recognition of pictures, objects, faces; Apraxias; Body-schema disturbance; right-left confusion; acalculia

* Tests such as AIIMS Neuropsychological Battery, PGI Battery of Brain Dysfunction, Dennis-Kaplan Executive Functions Test Battery, Behavioral Assessment of Dysexecutive Syndrome, etc., are some alternatives be used for obtaining a comprehensive profile. Similarly, tests such as the Montreal Cognitive Assessment Scale, SLUMS Exam, Frontal Assessment Battery, Cognitive Assessment Battery, etc., are some screening alternatives available in place of items from Addenbrooke’s Cognitive Exam. Tests such as the Mississippi Aphasia Screening Test, Indian Aphasia Battery developed at AIIMS, may also be used to evaluate language functions

**Table 2:** List of the tests commonly chosen for intra-operative evaluation at NIMHANS

Test	Domains	Test Description	Neuroanatomical Correlates
Orientation from MMSE	The performance in this test requires focused attention from the patient, comprehension of simple instruction, with demand on spoken language restricted to one word responses	It consists of 5 questions each testing varying degree of patient’s orientation time and place	Cortical and subcortical
Tests of comprehension from Mukundan’s battery	The performance in this test requires focused attention from the patient, with demand on spoken language increasing in complexity from one word responses to use of complex grammatical sentences.	It consists of questions that require auditory language comprehension. The questions are arranged in a graded manner of the demand of comprehension (simple to complex including emotional and abstract language comprehension).	Wernicke’s and corresponding cortical and subcortical regions (Posterior Peri-sylvian regions; Extra sylvian; inferior parietal (angular gyrus)
Western Aphasia Battery	The performance in this test requires focused attention from the patient, with simple to complex demand on comprehension and involving varying levels of language response, motor response as well as use of visual modality for reading and naming tasks.	It tests comprehension, fluency, spontaneity of spoken and written language.	Perisylvian regions anterior and posterior as well as extra sylvian watershed regions; inferior frontal; supplementary motor area; cingulate gyrus; angular gyrus.
Animal Names Test	The performance in this test requires focused and sustained attention, as well as language fluency.	The patient is required to generate as many novel names of animals as possible in 1 minute.	Ventral language regions; specifically anterior cingulate cortex, mid temporal, angular gyrus
Controlled Oral Word Association test	The performance in this test requires focused and sustained attention, as well as language fluency.	The patient is required to generate as many novel words as possible to a phonemic cue in 1 minute.	Anterior dorsal language pathways; inferior frontal regions; cingulate gyrus
Digit Span test	It is a test of verbal working memory; requiring the patient to respond verbally.	The patient is required to repeat an increasing span of numbers in two conditions-forward and backward.	Dorsolateral Pre-frontal cortex
Tests of naming	It requires the patient to perceive visual stimuli and respond verbally.	The patient can be asked to name a predetermined range of visual stimuli-common culturally common place pictures, objects, numbers, letters, colors, faces.	Ventral language regions; specifically anterior cingulate cortex, mid temporal, angular gyrus
Tests of repetition	lit requires auditory perception of verbal input and repetition verbally.	A component of Mukundan’s Battery,^[15]^ this test requires repetition of sentences, simple to complex.	Watershed regions
Tests of memory	It taps into the domains of verbal memory- encoding, recall as well as remote memory	It requires the patient to encode verbal input and repeat the same immediately as well as after a delay period; respond to questions of personal relevance. eg: memory items from Addenbrooke’s Cognitive Exam^[18]^	inferior frontal; cingulate gyrus; medial temporal; hippocampal and para-hippocampal
Tests of mental arithmetic	It taps into the domains of attention, working memory as well as calculation	It requires the patient to mentally calculate and respond to simple to complex questions of addition, subtraction, multiplication. Serial computation can also be given.	Dorsolateral prefrontal cortex; fronto parietal tracts; angular gyrus

## Key Message

Multidisciplinary team approach increases the efficacy of awake-craniotomy procedures. In this regard, it is important to operationalize a standard protocol for each discipline. This article aims to comment on operationalizing the role of clinical neuropsychologists in awake craniotomy.
